# Nuclear Receptors as Drug Targets in Cholestatic Liver Diseases

**DOI:** 10.1016/j.cld.2012.12.001

**Published:** 2013-05

**Authors:** Emina Halilbasic, Anna Baghdasaryan, Michael Trauner

**Affiliations:** a Division of Gastroenterology and Hepatology, Department of Internal Medicine III, Medical University of Vienna, Vienna, Austria; b Laboratory of Experimental and Molecular Hepatology, Division of Gastroenterology and Hepatology, Department of Internal Medicine, Medical University of Graz, Graz, Austria

**Keywords:** Cholestatic liver disease, Nuclear receptors, Cholestasis, Bile acids

## Abstract

Cholestatic liver diseases encompass a wide spectrum of disorders with different causes, resulting in impaired bile flow and accumulation of bile acids and other potentially hepatotoxic cholephils. The understanding of the molecular mechanisms of bile formation and cholestasis has recently improved significantly through new insights into nuclear receptor (patho)biology. Nuclear receptors are ligand-activated transcription factors, which act as central players in the regulation of genes responsible for elimination and detoxification of biliary constituents accumulating in cholestasis. They also control other pathophysiologic processes such as inflammation, fibrogenesis, and carcinogenesis involved in the pathogenesis and disease progression of cholestasis liver diseases.

## Key points

• Nuclear receptors (NRs) regulate ligand-activated transcription factor networks of genes for the elimination and detoxification of potentially toxic biliary constituents accumulating in cholestasis.
• Activation of several NRs also modulates fibrogenesis, inflammation, and carcinogenesis as sequels of cholestasis.
• Impaired NR signaling may be involved in the pathogenesis of cholestasis and genetic variants of NR-encoding genes are associated with susceptibility and progression of cholestatic disorders.
• NRs represent attractive targets for pharmacotherapy of cholestatic disorders, because their activation may orchestrate several key processes involved in the pathogenesis of cholestatic liver diseases.
• Several already available drugs may exert their beneficial effects in cholestasis via NR activation (eg, ursodeoxycholic acid via glucocorticoid receptor and pregnane X receptor; rifampicin via pregnane X receptor; fibrates via PPARα; budesonide via glucocorticoid receptor) and novel therapeutic developments target NRs (obeticholic acid - farnesoid X receptor).

## Introduction

Cholestasis may be best defined as an impairment of bile flow whereby bile reaches the duodenum in insufficient amounts.^1^ The cause of different cholestatic diseases is quite diverse, comprising hereditary and acquired diseases caused by genetic and environmental factors (discussed in previous articles in this volume). Independent of their cause, the main features of cholestatic liver disorders include an accumulation of cholephils such as bile acids (BAs) in the liver and systemic circulation.^2^ The accumulation of potentially toxic BAs leads to hepatocellular damage followed by inflammation and fibrosis, and, finally, depending on the disease severity and duration, may culminate in liver cirrhosis and hepatocellular or cholangiocellular cancer. To handle potentially toxic cholephils under physiologic and pathologic conditions, the liver possesses a complex network of nuclear receptor (NR)-regulated pathways that coordinate BA homeostasis and bile secretion to limit their concentrations and prevent hepatic as well as systemic accumulation. NRs are ligand-activated transcription factors that regulate a broad range of key hepatic processes^3^ in addition to hepatobiliary excretory function, such as hepatic glucose and lipid metabolism, inflammation, regeneration, fibrosis, and tumorigenesis.^4^ On activation by ligands, NRs change their conformation, which in turn facilitates the recruitment of coactivators and dissociation of corepressors and enables DNA binding and stimulation of gene transcription.^5^ The recruitment of cofactors fine tunes the regulation of transcription by NRs.^6^ The most relevant BA-activated NRs for regulation of hepatobiliary homeostasis, bile secretion, and, thereby understanding and treating cholestasis, include the farnesoid X receptor (FXR, NR1H4),^7^ pregnane X receptor (PXR, NR1I2),^8,9^ and vitamin D receptor (VDR, NR1I1).^10^ Apart from BAs, other biliary constituents such as bilirubin can also activate NRs, such as the constitutive androstane receptor (CAR, NR1I3). Furthermore, other nuclear receptors such as glucocorticoid receptor (GR, NR3C1) and fatty acid-activated peroxisome proliferator-activated receptors (PPARs), in particular PPARα (NR1C1) and PPARγ (NR1C3) as regulators of inflammation, fibrosis, and energy homeostasis, may also impact on biliary homeostasis and cholestatic liver injury. Because of their capability to control hepatic metabolism, NRs have emerged as promising therapeutic targets in many liver diseases, including cholestatic disorders. In this article, the principal role of NRs in the pathogenesis of various cholestatic disorders and how they may serve as drug targets in the management of cholestatic patients are discusssed.

## Nuclear BA receptor FXR and its biology

FXR has been identified as a main nuclear BA receptor,^7,11,12^ controlling synthesis and uptake of BAs as well as stimulating their elimination from liver. FXR is predominantly expressed in organs involved in BA transport and/or metabolism, such as liver, ileum, kidney, and adrenal glands.^13–15^ As many other NRs, it exerts its transcriptional activity by heterodimer formation with another NR retinoid X receptor (RXR, NR2B1).^13,16^ To initiate gene transcription, the FXR-RXR heterodimer binds to so-called inverted repeat 1 (IR-1) within the promoter sequence of target genes.^17^ Four FXRα isoforms coded as FXRα1-4 have been described,^18^ which have identical DNA-binding domain but may differ in gene regulation because of differences in ligand-dependent recruitment of coactivator/corepressor proteins, heterodimer formation with RXR, or DNA binding.^15,19,20^

**Fig. 1:**
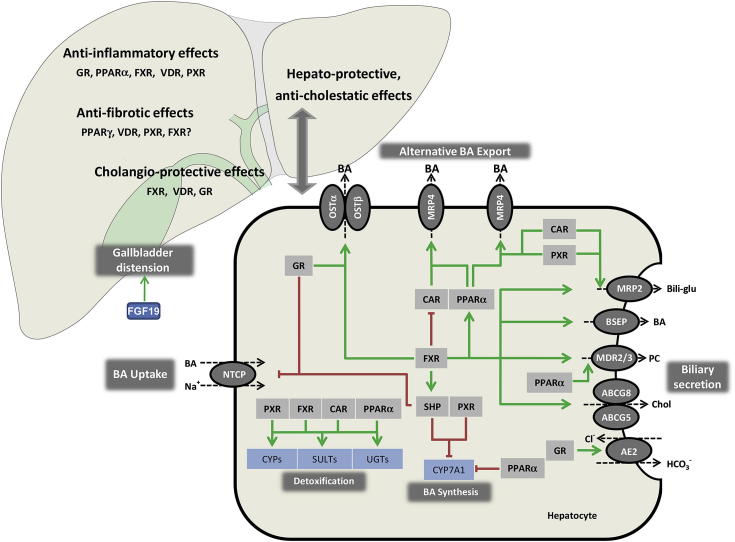
Role of nuclear receptors in maintaining hepatobiliary homeostasis. Activation of nuclear receptors (NRs) in hepatocytes ensures the balance between BA synthesis and detoxification, uptake, and excretion via regulation of expression of key hepatobiliary transporters. A network of negative feed-back and positive feed-forward mechanisms controls the intracellular load of biliary constituents, which may be hepatotoxic when they accumulate. BA-activated FXR is a central player in this network and represses (via GR in humans) hepatic BA uptake (NTCP) and (via SHP) BA synthesis (CYP7A1), promotes bile secretion via induction of canalicular transporters (BSEP, MRP2, ABCG5/8, MDR3), and induces BA elimination via alternative export systems at the hepatocellular basolateral (sinusoidal) membrane (OSTα/β). Several NR pathways converge at the level of CYP7A1 as a rate-limiting enzyme in BA synthesis. CAR and PXR facilitate adaptation to increased intracellular BA concentrations by upregulation of alternative hepatic export routes (MRP3 and MRP4) and induction of detoxification enzymes. PPARα regulates phospholipid secretion (via MDR3), but is also involved in detoxification pathways. Stimulation of AE2 expression by GR stimulates biliary bicarbonate secretion, thus reducing bile toxicity. Apart from regulating BA homeostasis, NRs have additional anti-inflammatory and anti-fibrotic effects. Their activation may result in induction of defensive mechanisms in bile duct epithelial cells. Green arrows indicate stimulatory effects and red lines indicate suppressive effects on target genes. AE, anion exchanger; BAs, bile acids; Bili-glu, bilirubin glucuronide; BSEP, bile salt export pump; CAR, constitutive androstane receptor; CYP7A1, cholesterol-7α-hydroxylase, CYPs, cytochrome P450 enzymes; FGF, fibroblast growth factor; FXR, farnesoid X receptor; GR, glucocorticoid receptor; MDR3, multidrug resistance protein 3, phospholipid flippase; MRP2, multidrug resistance-associated protein 2; MRP3, multidrug resistance-associated protein 3; MRP4, multidrug resistance-associated protein 4; NTCP, sodium taurocholate cotransporting polypeptide; OSTα/β, organic solute transporter α and β; PC, phosphatidylcholine; PXR, pregnane X receptor; PPARα, peroxisome proliferator-activated receptor α; PPARγ, peroxisome proliferator-activated receptor γ; SHP, small heterodimer partner; SULTs, sulfatation enzymes; UGTs, glucuronidation enzymes; VDR, vitamin D receptor.

The central role of FXR encompasses the regulation of the enterohepatic circulation and intracellular load of BAs (Fig. 1). By inhibition of the basolateral uptake transporter sodium/taurocholate cotransporting polypeptide, solute carrier family 10, member 1 (NTCP; SLC10A1) and upregulation of the canalicular export transporter bile salt export pump (BSEP; ABCB11) in hepatocytes, FXR reduces hepatocellular BA levels by limiting their uptake from the sinusoidal blood and promoting their biliary excretion (see Fig. 1).^21–24^ In addition, FXR reduces endogenous BA synthesis via classical and alternative pathways through the inhibition of rate-limiting enzymes CYP7A1, CYP8B1, and CYP27A1 (reviewed in^25^) (see Fig. 1). The molecular mechanism underlying the inhibitory effects of FXR are linked to FXR-mediated induction of an atypical NR short heterodimer partner (SHP; NR0B2) and which acts as transcriptional repressor because of interference with other NRs such as liver X receptor (LXR, NR1H3), liver receptor homolog 1 (LRH-1, NR5A2), and hepatocyte nuclear factor 4α (HNF4α, NR2A1).^26–29^ Additional important regulatory mechanisms for inhibition of BA synthesis include FXR-mediated induction of the intestinal hormonelike peptide fibroblast growth factor (FGF19; in rodents Fgf15), which reaches the liver via portal blood and binds to its specific receptor fibroblast growth factor receptor 4, resulting in activation of intracellular JNK pathway to inhibit CYP7A1 gene expression.^30–32^ As a target of FXR, FGF19 (Fgf15) represents a hormone that signals after food intake via the gut liver axis, suppressing the BA synthesis, inducing gallbladder relaxation and refilling,^33^ mediating (insulin-independent) insulin-mimetic effects such as stimulation of glycogen and protein synthesis and inhibition of gluconeogenesis,^34^ while unlike insulin, suppressing the lipogenesis.^35^ As such, FGF19 as an FXR target gene also represents an interesting target of anti-diabetic therapy.^36^

The role of FGF19 in cholestasis is yet to be elucidated. Although FGF19 is not expressed in hepatocytes and systemic FGF19 under physiologic conditions originate from the intestine, its hepatocellular expression is highly induced in cholestasis.^37^ Furthermore, FGF19 is highly expressed by human gallbladder epithelium and is secreted to the bile especially after treatment with FXR ligands.^38^ Because BAs may induce mucin production via FXR in gastric epithelial cells,^39^ it is attractive to speculate that BA-FXR-FGF19 signaling cascade may protect biliary epithelia against detergent BAs via mucin secretion.

Apart from repression of BA synthesis, FXR is able to induce alternative basolateral BA transport through organic solute transporter α/β (OSTα/β)^40,41^ and detoxification through transcriptional induction of hydroxylation enzyme CYP3A1, sulfo-conjugation by sulfatation enzymes 2A1 (SULT2A1), and glucuronidation by glucuronidation enzyme (UGT2B4) as additional potent mechanisms protecting hepatocytes from BA toxicity (reviewed in^3,42^) (see Fig. 1).

Biliary BAs are normally present in the form of mixed micelles together with phospholipids and cholesterol. Importantly, hepatic FXR promotes bile secretion not only through regulation of BA export but also via induction of canalicular phopholipid floppase MDR3 (Mdr2 in rodents)^43^ and human canalicular bilirubin conjugate export pump multidrug resistance protein 2 (MRP2; via a hormone response element ER-8) (see Fig. 1).^44^ The regulatory role of FXR in secretion of biliary phospholipids (and perhaps even glutathione) may be critical for the protection of hepatocytes' canalicular membrane as well as the apical membrane of bile duct lining cells against the detergent properties of secreted BAs.

In addition to BAs as principal endogenous FXR ligands, an intermediate product of BA synthesis oxysterol 22(*R*)-hydroxycholesterol and androsterone has been identified as endogenous FXR activators.^45,46^ Furthermore, several other natural substances have been recognized to exert agonistic or antagonistic effects on FXR. For example, stigmasterol, a compound present in soy-derived lipid emulsions used for total parenteral nutrition, showed FXR antagonistic activity, probably contributing to the total parenteral nutrition–induced cholestasis by inhibiting its target genes BSEP, FGF19, and OSTα/β.^47^

## FXR in cholestatic liver diseases

Because FXR is a central regulator of bile formation and BA homeostasis, one might expect that dysregulation or dysfunction of FXR may play a key role in the pathogenesis of cholestasis. However, FXR variants have been identified in only a few cholestatic syndromes^48–50^ and FXR may rather orchestrate secondary adaptive responses to cholestasis. Among progressive familiar intrahepatic cholestasis (PFIC) syndromes, only PFIC1 patients showed reduced hepatic and ileal FXR levels.^49,50^ Acquired cholestatic conditions, such as drug-induced liver injury and intrahepatic cholestasis of pregnancy (ICP), have also been associated with FXR dysfunction. In drug-induced liver injury and ICP, drug-mediated and hormone (metabolite)-mediated inhibition of hepatobiliary transporters may contribute to the pathogenesis.^51^ A common FXR genetic variant FXR1*B was associated with reduced gene expression of hepatic target genes SHP and organic anion transporting polypeptide 1B3 (OATP1B3),^52^ a sinusoidal transporter that mediates the uptake of several drugs and peptides such as cholecystokinin and digoxin.^53,54^ These findings indicate that FXR dysfunction may largely influence the pharmacokinetics and pharmacodynamics of various drugs, thus significantly contributing to drug response as well as severity of potential side effects and therapeutic outcomes in affected patients.

FXR may play a role in gallstone disease because FXR knockout mice show biliary cholesterol supersaturation, formation of cholesterol crystals, and increased bile salt hydrophobicity, whereas synthetic FXR agonist GW4064 efficiently reduced gallstone formation in mice.^55^ In contrast to these findings, no common polymorphism has been identified in patients with gallstone disease from different ethnic groups. However, an FXR variant was associated with gallstone prevalence in Mexican patients.^56^ Interestingly, patients with gallstones showed repressed expression of PGC1α,^57^ a transcriptional coactivator of FXR^58,59^ that may additionally induce FXR gene transcription via PPARγ and HNF4α.^59^ Thus, it is plausible to speculate that peroxisome proliferator-activated receptor gamma, coactivator 1 alpha (PGC1α)-associated reduction of FXR activity could contribute to altered bile composition and gallstone formation through inhibition of BSEP and MDR3. However, larger cohorts and more standardized sample analysis are required to draw conclusive statements regarding the role of FXR in human gallstone disease.

In chronic cholestatic liver diseases (eg, primary biliary cirrhosis [PBC] and primary sclerosing cholangitis [PSC]) prolonged duration of cholestasis may induce adaptive, secondary changes in transporter expression self-protective mechanisms of hepatocytes against retaining cholephils. For example, in PBC patients, repression of BA uptake systems (NTCP, OATP2) together with induction of basolateral efflux systems (MRP3, MRP4, and OSTα/β) support the elimination of retained BAs from the liver as cholestasis progresses with advanced disease.^41,60–65^ Experimental studies in rodents have uncovered a complex interplay of several regulatory pathways under control of FXR and other NRs that are activated by accumulating biliary constituents mediating these transporter changes.^42^ However, these intrinsic defense mechanisms are not sufficient to rescue the liver from cholestatic injury, because chronic cholestasis induces fibrosis and ultimately cirrhosis occurs, and additional pharmacologic activation may represent a mechanism of counteracting cholestasis by enhancing these intrinsic adaptive mechanisms as delineated below.^66^

An increasing body of evidence suggests that BA and FXR signaling regulates liver cell growth. Mice lacking FXR as well as mice lacking its downstream target SHP develop hepatocellular cancer (HCC).^67–69^ Downregulation of SHP has also been observed in human HCC.^70^ Notably, an increased risk for HCC has been observed in children with PFIC resulting from deficiency of the FXR target BSEP,^71^ further underlining the carcinogenic potential of BAs in liver. A weakened defense against potential carcinogenic BAs, subsequent hepatic inflammation, together with the absence of direct regulatory effects on the cell cycle, may explain the carcinogenic potential resulting from loss of FXR and SHP.^69,72,73^ A direct role of FXR on cell proliferation and apoptosis is underlined by the fact that not only does FXR play a crucial role in hepatocellular cancer, but also its alterations have also been implicated in colorectal and breast carcinogenesis.^74,75^

## Therapeutic potential of FXR in cholestasis

In the last several years, various BA-derived or non-BA-based FXR activators have been developed as potential therapeutics against cholestasis. The protective effects of FXR were demonstrated in several animal models. A non-BA synthetic FXR agonist GW4064 and BA-derived 6α-ethyl derivative of chenodeoxycholic acid (6E-CDCA or INT-747 or obeticholic acid; OCA) have beneficial effects in mouse models of chemically induced liver injury (α-naphthylisothiocyanate (ANIT) and estradiol-induced) or in bile duct-ligation (BDL).^76,77^

Recently 3 BA-based therapeutic compounds were compared in Mdr2 (mouse ortholog of human phospholipid export pump MDR3) knockout mice, a model of bile duct injury and biliary fibrosis associated with the toxic bile composition caused by absent biliary phospholipids^78^: a selective FXR ligand (INT 747), a selective ligand (INT-777) for TGR5 (another G protein coupled BA receptor located at the plasma membrane), and dual ligand for FXR and TGR5, with strong FXR agonistic properties (INT-767). Only INT-767 with dual agonistic in vitro activity toward FXR and TGR5 improved serum liver tests, portal inflammation, and biliary fibrosis. This compound induced bile flow and biliary bicarbonate output with simultaneous reduction of biliary BA output in wild-type but not in FXR-deficient mice, emphasizing the role of FXR (but not TGR5) in mediating these effects. The underlying mechanisms seem to include FXR-dependent induction of carbonic anhydrase 14, a hepatocellular membrane-bound enzyme that may promote bicarbonate transport due to formation of a functional complex with bicarbonate transporter anion exchanger 2 (AE2).^79^ These results uncovered an important role of FXR in regulation of biliary bicarbonate secretion protecting against intrinsic BA toxicity. Notably, the (weaker) selective FXR agonist INT-747 deteriorated liver injury in the Mdr2 knockout mice and the selective TGR5 agonist had no therapeutic effect, showing a minor role of biliary TGR5 for bile duct injury in this mouse model.

In addition to hepatocytes, cholangiocytes also play an important role in bile formation. Importantly, FXR is also expressed in human biliary epithelium, where it may play a critical role in ductular bile generation by alkalinization and fluidization through secretory mechanisms known to be predominantly regulated by complex neuro-endocrine as well as local mechanisms.^80^ The potential role of FXR in secretory function of biliary epithelium became apparent when endogenous FXR agonist CDCA as well as non-BA FXR agonist GW4064 induced gene expression of vasoactive intestinal polypeptide receptor 1 (VPAC-1),^80^ a receptor of vasoactive intestinal polypeptide in human gallbladder. Because vasoactive intestinal polypeptide acts as a very potent secretagogue^81^ in cholangiocytes, FXR-mediated VPAC-1 induction indicates a potential role for FXR in regulating the BA-independent bile flow in biliary epithelium.

In addition, CDCA (a potent endogenous FXR ligand) is able to induce expression of cathelecidin, the major anti-microbial peptide known to counteract the LPS, in human cholangiocytes, suggesting that BAs/FXR might play an important role in sterility of the biliary tree and protection against bile duct inflammation.^82^ In fact, the observation that FXR-deficient mice showed increased baseline hepatic inflammation and are more prone to LPS-induced liver injury^67,83^ suggests a direct anti-inflammatory role of FXR, which has been be explained via direct interference with the nuclear factor kappa-B (NF-κB).^83^ Notably, this effect is not only hepatocyte-specific but also was reported in vascular smooth muscle cells.^84^ The anti-inflammatory effects of FXR are further supported by induction of suppressor of cytokine signaling 3 that inhibits STAT3 signaling.^85^ Notably the anti-inflammatory effects of FXR are not liver-specific, but were also demonstrated in intestine, where INT-747 reduced intestinal inflammation and permeability in experimental models of colitis.^86^ Because bacterial overgrowth and increased intestinal permeability may play an important role in the pathogenesis of ascending biliary inflammation and cholestasis, a tight control of intestinal bacterial flora is likely to be protective in cholestasis. Bacterial overgrowth was successfully reversed by the oral BA supplementation in a rat model of intestinal BA depletion,^87,88^ findings that together with prevention of postoperative endotoxemia by preoperative administration of sodium deoxycholate in patients with obstructive cholestasis^89^ provide evidence for a role of intestinal BAs/FXR in maintaining the normal bacterial flora and gut integrity. Indeed, bacterial overgrowth and intestinal injury were decreased in the BDL model of obstructive cholestasis by GW4064 in an FXR-dependent manner^90^ and selective intestinal FXR-overexpression reduced liver injury by decreasing the BA pool size and hydrophobicity as well as improving the intestinal permeability in BDL and ANIT-induced liver injury.^91^ Moreover, FGF19 treatment protected mice from CBDL-induced liver injury, whereas selective intestinal FXR overexpression decreased liver injury in the genetic Mdr2 knockout mouse model of cholestasis, confirming the importance of intestinal FXR for liver disease.^91^ Taken together, FXR ligands counteract hepatic inflammation at several levels: directly via interaction with inflammatory pathways in hepatocytes as well as in non-parenchymal hepatic cells and by reducing release of inflammatory mediators from the intestine via a decrease in intestinal permeability and bacterial translocation. The latter may be of particular interest for the treatment of obstructive cholestasis with collapse of gut integrity and cholestatic liver disease associated with inflammatory bowel disease such as PSC.

Although many cholestatic liver diseases progress to liver fibrosis and finally cirrhosis, the question of whether FXR affects the fibrogenesis still remains unclear. Interestingly, FXR was also shown to have direct anti-fibrotic effects in hepatic stellate cells (HSCs) via activation of SHP.^92,93^ However, another study showed very low or no FXR and SHP expression in human HSCs and murine periductal myofibroblasts,^94^ suggesting indirect anti-fibrotic effects.

Collectively, FXR activation by endogenous or synthetic agonists represents an efficient mechanism to counteract cholestasis by a synchronized network of hepatoprotective mechanisms: (1) reducing intrahepatic BA load via repression of BA synthesis and an increase in BA export (via BSEP on the canalicular and OSTα/β on the basolateral membrane); (2) changing bile composition at the hepatocellular level (by increasing relative phospholipid and bicarbonate secretion), ultimately resulting in a less toxic bile protecting hepatocytes and cholangiocytes; (3) impacting on ductular bicarbonate secretion (via induction of VPAC-1); (4) mediating direct anti-inflammatory effects in hepatocytes (via inhibition of NF-κB and STAT3) and non-parenchymal liver cells; (5) impacting on the gut-liver axis (by induction of FGF19, a suppressor of BA synthesis and by reducing a bacterial overgrowth and intestinal permeability in obstructive cholestasis).

Because targeted FXR activation has been recognized as a promising therapeutic option for patients with cholestasis, FXR agonists have already entered the clinical trials. Specifically, combination therapy of ursodeoxycholic acid (UDCA) with the INT-747 in phase II clinical trials in PBC patients not responding to UDCA showed substantial reduction of biochemical parameters of liver damage and cholestasis, such as ALT and ALP, after short-term and long-term administration.^95,96^ In line with the results obtained with combination therapy, INT-747 monotherapy in PBC patients also achieved a significant reduction of serum markers of liver damage and cholestasis after 12 weeks of treatment.^97^ Dose-dependent itching was reported to be the most common adverse event in patients receiving higher doses of INT-747. Because pruritus represents a common symptom of PBC that may lead to severe disability in suffering patients, subsequent clinical trials have excluded patients suffering from pruritus because of the disease. The results of a multicenter, placebo-controlled, randomized phase III clinical trial, testing INT-747 in PBC patients who have not non-responded to standard UDCA, are eagerly awaited.

## Nuclear xenobiotic receptors PXR and CAR and their biology

The primary function of PXR and CAR is to regulate genes responsible for the detoxification and elimination of a broad spectrum of potentially toxic endogenous and exogenous compounds.^98–100^ To achieve their detoxifying function and to protect from various xenobiotics, both PXR and CAR act as low-affinity, broad-specificity xenosensors, which are activated by a broad range of structurally unrelated compounds (eg, rifampicin, clotrimazole, synthetic steroids such as 5β-pregnane-3, 20-dione, pregnenolone 16α-carbonitrile (PCN), dexamethasone, anti-depressant St. John’s wort).^100–103^ Apart from xenobiotics, also potentially toxic endogenous compounds such as BAs^8,9^ and bilirubin^104^ can activate PXR and CAR. After their activation, PXR and CAR coordinately induce a machinery of genes responsible for detoxification and elimination of their activating toxic ligands.

Various enzymes involved in phase I (catalyzing hydroxylation) and phase II (catalyzing glucuronidation and sulfatation) detoxification as well as many drug transporters are target genes of PXR and CAR, converting their substrates into more hydrophilic and therefore less toxic and easier cleared compounds (see Fig. 1).^98,100,105^ In cholestatic condition, the activation of PXR and CAR may be beneficial because PXR, as a BA-activated receptor, is also responsible for basal repression of CYP7A1 as a rate-limiting enzyme for BA synthesis,^8^ and both PXR and CAR are inducers of BA detoxification enzymes such as CYP3A4 (Cyp3a11 in mice), Cyp2b10, and SULT2A1 (see Fig. 1).^106^ Furthermore, they activate the transcription of UGT1A1, a key enzyme for bilirubin glucuronidation (see Fig. 1).^9^ Finally, PXR has been identified as an FXR target gene,^107^ suggesting an evolutionary-based cross-talk between BA-activated NRs in the protection against BA toxicity.

## PXR and CAR in cholestatic liver diseases

Altered function of PXR and CAR is involved in both pathogenesis and adaptation to cholestatic liver disease. Genetic variants of PXR are associated with increased susceptibility for ICP, as well as with lower neonatal weight and Apgar score in South American populations.^108^ In contrast, PXR variants were not found to be associated with ICP in a Caucasian population, but it should be emphasized that this study considered only coding sequence and no regulatory promoter regions were examined.^109^ Furthermore, PXR polymorphisms have been associated with the disease course in PSC.^110^

In patients with obstructive cholestasis, a pronounced increase in PXR and CAR expression is observed, followed by an increase in their target genes (MRP3 and MRP4),^111,112^ consistent with activation of self-protective pathways in cholestatic hepatocytes (see Fig. 1). The role of PXR and CAR for limiting the progression of liver injury in cholestasis was confirmed by reduced expression of these NRs in late-stage cholestasis in children suffering from biliary atresia,^113^ and low PXR and CAR expression were associated with poor prognosis in these patients. In PBC, a moderate reduction of PXR and CAR expression levels was observed.^66^ The involvement of PXR and CAR in fibrogenic processes was further underlined by their low expression in hepatitis C patients with advanced fibrosis.^114^ Of note, neonates have low hepatic expression of CAR, as the main NR coordinately regulating bilirubin clearance, thus providing a possible explanation for their higher susceptibility to (neonatal) jaundice.^104^

## PXR and CAR as therapeutic targets

Because of their central role in BA detoxification and transport, PXR and CAR represent attractive targets for drug therapy of cholestasis. Ligands for both receptors have already been used to treat cholestasis and pruritus, long before their mode of action has been fully understood. As such, rifampicin is a classic ligand for PXR and not only is effectively used to treat pruritus but also improves liver function tests in PBC, compatible with a direct anti-cholestatic effect.^115–117^ In the otherwise healthy gallstone patients, rifampicin enhanced BA detoxification as well as bilirubin conjugation and excretion through induction of CYP3A4, UGT1A1, and MRP2, thereby decreasing bilirubin and deoxycholic acid concentrations in serum as well as lithocholic (LCA) and deoxycholic acid concentrations in bile.^118^ The potential mechanisms by which rifampicin improves cholestatic pruritus have recently been further expanded by linking its action to the lysophospholipase autotaxin and its product, lysophosphatidic acid, as potential mediators of cholestatic pruritus.^119^ Notably PXR inhibits autotaxin expression, which may add to the anti-pruritic action of rifampicin.^120^

Phenobarbital was also given to patients long before the identification of CAR as its molecular target.^115,121,122^ Notably, 6,7-dimethylesculetin, a compound present in Yin Chin used in Asia to prevent and treat neonatal jaundice, accelerates bilirubin clearance by activation of CAR.^123^ Activation of CAR increases hepatic expression of the bilirubin-clearance pathway, including the induction of bilirubin glucuronyl transferase, a key enzyme of bilirubin glucuronidation and canalicular bilirubin-glucuronide export pump MRP2.^104,123^ In addition to CAR as prototypic bilirubin-activated receptor, PXR also promotes bilirubin detoxification and clearance via induction of its glucuronidation and export.^44,124^

In a rodent model, pharmacologic stimulation of PXR counteracted LCA-induced liver toxicity by induction of Cyp3a11 (CYP3A4 in human) and SULT2A1, both involved in BA detoxification.^8,9^ Similarly, administration of PXR ligands reduced liver injury, bilirubin, and BA levels in CA-fed mice via induction of Cyp3a11 and MRP3.^125^ LCA-induced hepatotoxicity was also diminished by pharmacologic activation of CAR, mediating a shift in BA biosynthesis toward the formation of less toxic BAs, as well as a decrease in hepatic bile acid concentrations.^126^ In obstructive cholestasis (BDL) in mice, administration of PXR and CAR ligands reduced serum parameters of cholestasis (ie, bilirubin and serum BA levels) by induction of phases I and II detoxification and transport systems.^127^ However, elevated liver enzymes in these animals point out potential hepatotoxic side effects of the used substances and concentrations, at least under conditions when bile flow is completely blocked.^127^ However, pharmacologic stimulation of PXR and CAR could be therapeutically superior to activation of FXR in obstructive cholestasis, because stimulation of these xenobiotic sensors lacks potentially negative effects associated with stimulation of bile flow. This precaution is also underlined by the fact that FXR stimulation may lower the induction of MRP4 by CAR ligands, thereby limiting the main alternative BA export route from cholestatic hepatocytes.^128^

Apart from its anti-cholestatic effects, PXR also has anti-fibrotic and anti-inflammatory properties that may be beneficial in complex cholestatic liver diseases such as PSC and PBC. PXR stimulation in human HSC inhibits their transdifferentiation to fibrogenic myofibroblasts, inhibits expression of the major profibrogenic cytokine TGF-1β, and markedly slows proliferation.^129^ In mice, PCN, a potent activator of rodent PXR, inhibited carbon tetrachloride–induced fibrosis in a PXR-dependent manner.^130^ In addition, activation of PXR inhibited endotoxin-induced NF-κB activation and cytokine production, and mice lacking PXR have higher susceptibility to inflammatory agents.^131,132^ Suppression of humoral and cellular immune response by rifampicin has been recognized 40 years ago^133^ and may now at least in part be explained by ligand-induced SUMOylation of PXR subsequently repressing NF-κB target genes.^134^

Finally, PXR is essential for liver regeneration because mice lacking PXR have impaired hepatocyte proliferation.^135^ Activation of CAR also induces a strong proliferative response in mouse liver by stimulating cyclin D1,^136^ which is mandatory for cell-cycle progression in proliferating hepatocytes, suggesting that CAR agonists could also be potentially useful to stimulate hepatocyte proliferation after liver resection. However, CAR activation also plays a key role for liver tumor promotion in phenobarbital-treated mice.^137,138^

Collectively, pharmacologic stimulation of PXR and CAR in chronic cholestatic liver disease may improve the disease course via at least 4 potential beneficial mechanisms: (1) repression of BA synthesis and increase in BA and bilirubin detoxification and elimination pathways, which will enhance the ability of the liver to reduce levels of toxic cholephils; (2) suppression of inflammation and fibrosis; (3) promotion of hepatocellular regeneration; and (4) amelioration of pruritus. However, it must be emphasized that both PXR and CAR ligands are potentially hepatotoxic and carcinogenic; therefore, novel compounds targeting PXR and CAR with fewer side effects need to be developed.

## VDR and its biology

The main function of VDR is to mediate the effects of its natural ligand calcitriol (1α, 25-dihydroxyvitamin D3 [1,25-VitD3]) on calcium homeostasis, but VDR also regulates cell proliferation and differentiation and has immunomodulatory as well as anti-microbial functions.^139^ Importantly, VDR is also an intestinal sensor for secondary BAs and as such is activated by lithocholic acid.^10^ In the liver, VDR is not expressed in hepatocytes, whereas other non-parenchymal liver cells such as Kupffer cells, endothelial cells, biliary epithelial cells, and HSCs show considerably high levels of expression.^140^ In bile duct epithelial cells, activation of VDR by BAs or vitamin D induces cathelicidin expression, which is an anti-microbial peptide known to be protective against bacterial infection,^82^ thus contributing to innate immunity in the biliary tract. In HSCs VDR is highly expressed in the quiescent state and its expression decreases during activation. Stimulation of VDR in activated HSCs inhibits their proliferation and suppresses collagen production, explaining the anti-fibrotic effects of vitamin D supplementation in the rat model for liver fibrosis.^141^ In the intestine, stimulation of VDR increases the expression of human and rodent apical sodium/bile acid transporter (ASBT),^142^ an ileal BA uptake transporter, and of MRP3, a basolateral BA export pump, in mouse colon.^143^ In the liver, despite low expression of VDR in hepatocytes, treatment with VDR agonists stimulate BA detoxification enzymes (such as SULT2A1 and Cyp3a11, a mouse homolog of human CYP3A4).^10,144,145^ Whether VDR may have beneficial effects on BA-induced hepatocellular injury is difficult to predict because of reported negative interactions of VDR with FXR and inhibition of FXR transactivation by 1,25-VitD3 in vitro.^146^

## VDR and cholestatic liver diseases

Multiple polymorphisms in the coding sequence and promoter region of VDR may alter the immune response and specific VDR variants are associated with several immune-mediated liver diseases. As such, VDR polymorphisms are associated with susceptibility and clinical appearance of PBC^147–151^ and autoimmune hepatitis.^147,149^

Because impaired absorption of fat-soluble vitamins is a hallmark of cholestasis and severe liver dysfunction, low serum vitamin D levels are commonly observed in patients with cholestasis and may alter VDR activity with consequences beyond bone metabolism. Low 1,25-VitD3 levels impair fetal outcome (inversely correlating with meconium staining) in patients with ICP.^152^ VDR expression in bile duct epithelial cells was inversely correlated with steatosis, lobular inflammation, and NAS score in patients with non-alcoholic fatty liver disease.^153^ A growing body of evidence suggests that vitamin D signaling plays a role in the progression of fibrosis in various liver diseases, including fatty liver disease and hepatitis C,^141^ and development of cancer,^154,155^ including HCC,^156^ but data for cholestatic liver diseases in this context are still limited. VDR expression in primary rat HSCs decreases on activation of these cells, whereas 1,25-VitD3 inhibits proliferation, decreases expression of profibrogenic, and increases expression of anti-fibrotic genes.^141^

Accumulation of LCA during cholestasis decreases the effects of vitamin D on human osteoblasts, acting as a competitive ligand for VDR^157^ and thereby promoting osteoporosis in cholestatic patients. Interestingly, vitamin D supplementation was also associated with lower fatigue appearance in patients with PBC,^158^ suggesting a potential link between vitamin D deficiency and this disabling symptom in cholestasis. Further studies will have to show whether this may be linked to muscular effects of vitamin D.

## VDR as therapeutic target

According to the predominance of VDR in non-parenchymal liver cells, activation of VDR in the liver has mainly anti-inflammatory and anti-fibrotic effects that may be beneficial in chronic cholestatic liver disease (such as PBC and PSC). “Classical” targeting of VDR through vitamin D substitution improves bone density in patients where cholestasis leads to chronic vitamin D deficiency and increased rates of osteoporosis. The anti-fibrotic potential of VDR stimulation was confirmed by reduced fibrosis in a rat model of liver fibrosis.^141^ Furthermore, treatment with 1,25-VitD3 suppressed the production of pro-inflammatory cytokines in the liver of BDL mice,^159^ underlining the potential of VDR ligands to prevent cholestasis-induced inflammatory response. These anti-inflammatory and anti-fibrotic effects of vitamin D suggest that vitamin D supplementation could have additional therapeutic effects in patients with PBC and PSC beyond the rationale for preventing and treating hepatic osteodystrophy. However, the rather complex role of VDR in regulation of BA uptake in intestine and regulation of BA metabolism in liver as well as its negative effects on FXR must be considered also. Although the use of vitamin D or synthetic VDR agonist as disease-modifying agents represents an attractive therapeutic concept for cholestatic liver diseases, especially when vitamin D levels are already low because of cholestasis, data from controlled studies are lacking.

## PPARs and their biology

PPARα, PPARγ, and PPARδ are 3 structurally homologous receptors and are activated by endogenous fatty acids and their derivatives to control important metabolic pathways in lipid and energy homeostasis.^160–162^ PPARα is highly expressed in tissues with active fatty acid catabolism, such as liver, heart, kidney, brown adipose tissue, muscle, small intestine, and large intestine; PPARγ is expressed mainly in adipose tissue and in the immune system and PPARδ is ubiquitously expressed.^163,164^ PPARα controls energy expenditure and catabolic metabolism by inducing β-oxidation, whereas PPARγ is critical for adipocyte differentiation and energy storage by adipocytes mediating anabolic energy state.^165,166^

Besides its role in the regulation of fatty acid metabolism, PPARα is involved in BA homeostasis. Fibrates, which are PPARα activators, induce the expression of phase II enzymes SULT2A1, UGT2B4, and UGT1A3 as well as ASBT, BA uptake transporter, in cholangiocytes and enterocytes.^167–170^ Furthermore, PPARα represses BA synthesis by reducing HNF4α binding to the CYP7A1 promoter (see Fig. 1).^171–174^ PPAR ligands such as fibrates repress BA synthesis and promote phospholipid secretion into bile,^173,174^ via induction of MDR3,^175^ thus counteracting the aggressive biliary BA milieu (see Fig. 1).

In contrast to PPARα, a direct role for PPARγ in the regulation of BA metabolism has not yet been reported, probably because of a low expression pattern in hepatocytes. Targeting PPARγ is of particular interest for inflammatory cholestasis because of its crucial role in attenuation of inflammation-mediated transporter and enzyme changes. In the LPS model of inflammatory cholestasis treatment with glitazones, as synthetic PPARγ ligands and accepted anti-diabetic drugs, attenuated repression of NTCP, BSEP, and Cyp3a11, without affecting cytokine levels via inhibition of RXRα, export from the nucleus.^176^ In addition, PPARγ represses transcriptional activation of inflammatory response genes as a negative regulator of cellular toll-like receptor signaling in inflammatory cells as well as in cholangiocytes.^177^ Moreover, in HSCs, PPARγ regulates their activation and has profound anti-fibrotic effects modulating the wound-healing process by amelioration of inflammation, oxidative stress, and matrix remolding in the injured liver.^178^

## PPARs and liver diseases

PPARγ is involved in inhibition of inflammation and production of pro-inflammatory cytokines. Because bile duct destruction in PBC is Th1 cytokine mediated, it may not be surprising that PPARγ expression, which is high in normal bile ducts, is reduced in damaged bile ducts and may be associated with the Th1-predominant milieu and favor the development of chronic cholangitis in PBC.^179^ Immune modulation using PPARγ ligands may be of therapeutic benefit to attenuate biliary inflammation in PBC. In HSCs from BDL mice developing biliary cirrhosis, PPARγ expression and DNA binding was dramatically reduced, demonstrating that HSC activation is associated with the reductions in PPARγ expression.^180^

## PPARs as therapeutic targets

The effects of PPARα on biliary phospholipid secretion, BA metabolism, and synthesis make PPARα an interesting therapeutic target in the treatment of cholestasis. One of the key rationales for a beneficial role of fibrates in cholangiopathies may be upregulation of MDR3^181^ and its subcellular redistribution toward the canalicular membrane,^182^ thereby increasing the biliary content of phosphatidylcholine and reducing the aggressive potential of BAs in bile, subsequently protecting the biliary tree. This concept is supported by findings in patients undergoing percutaneous transhepatic biliary drainage, who showed increased biliary phospholipid secretion after treatment with bezafibarte,^183^ although the same study reported that patients with PBC had already increased MDR3 expression that was not further upregulated by bezafibrate treatment. Moreover, treatment with bezafibrate may have additional anticholestatic effects as supported by repression of BA synthesis (CYP7A1 and CYP27A1) and BA uptake (NTCP) and increased BA detoxification enzyme CYP3A4 in human hepatoma cell lines.^184^ Repression of BA synthesis and increased detoxification of BA by fibrates were confirmed in early-stage PBC patients measuring reduction of 7α-hydroxy-4-cholesten-3-one (C4), a marker of BA synthesis, and an increase of 4β-hydroxycholesterol, a marker of CYP3A4/5 activity after bezafibrate and UDCA combination therapy in comparison to UDCA monotherapy.^184^ Finally, the anti-inflammatory effects of PPARα could also add to potential beneficial effects in cholestasis.

Clinically the beneficial effects of PPAR ligands in cholestasis were recognized for more than a decade and multiple pilot studies have evaluated their therapeutic effectiveness in patients with PBC. More than a dozen uncontrolled pilot trials using bezafibrate and fenofibrate showed beneficial effects on biochemical parameters and in part also on histologic findings in patients with PBC.^184–200^ Some of these studies have tested the fibrates as monotherapy in comparison to UDCA monotherapy, but most were designed to test their effects in patients with partial or absent UDCA response by add-on therapy with either fenofibrate or bezafibrate. All these pilot studies showed the benefit of combination therapy. However, no placebo-controlled randomized studies have been performed so far and such studies are urgently needed before implementing UDCA/fibrate combination therapy as standard for PBC patients with suboptimal response to UDCA. However, one should be aware that fibrates increase the risk for gallstone formation,^201^ a side effect that could be linked to suppression of BA synthesis and that may represent a potential limitation for treatment in patients with biliary damage and an already increased susceptibility to gallstone formation such as PBC.

Moreover, PPARα ligands may also be beneficial in patients with chronic hepatic graft-versus-host disease of the liver. A combination of UDCA and bezafibrate therapy in this patient population significantly improved liver biochemistry after 1 month of treatment.^202^ Long-term clinical trials are also needed.

Other hypolipidemic drugs, such as inhibitors of 3-hydroxy-3-methylglutaryl-coenzyme A reductase (statins), are indirect activators of PPAR, also have pleiotropic anti-inflammatory effects,^203^ and stimulate phospholipid secretion by induction of Mdr2.^204,205^ Statins have also been tested in the treatment of PBC. Although initial smaller studies suggested improvement of cholestasis under statin treatment,^206–208^ a recent dose finding study was unable to demonstrate improvement of cholestasis in PBC patients with an incomplete response to UDCA.^209^

In addition to PPARα, PPARγ activation may also be effective in cholestatic diseases, in particular by ameliorating fibrosis and inflammation, thus limiting disease progression. The inhibitory effects of PPARγ ligands on collagen synthesis in HSCs^180^ were also observed in a model of obstructive cholestasis (BDL) where treatment with troglitazone inhibited ductular reaction and fibrosis.^210^ However, troglitazone, a PPARγ ligand, was meanwhile withdrawn from the market because of hepatotoxicity and no experimental or clinical data on other glitazones are available.^211,212^ The plant extract curcumin, the yellow pigment of the spice turmeric, also targets PPARγ. Notably, natural compounds such as curcumin inhibited inflammatory activation of cholangiocytes and activation of portal myofibroblasts in a PPARγ-dependent manner, ameliorating biliary fibrosis in various animal models.^213,214^

## GR and its biology

Glucocorticoids are natural ligands of GR. GR is expressed in most human cells and plays a role in numerous metabolic pathways including carbohydrate and protein homeostasis, mediates negative feedback on the hypothalamic–pituitary–adrenal axis, and has strong anti-inflammatory and immunosuppressive effects.^215^ Apart from regulating systemic response to stress, GR and glucocorticoids also regulate BA homeostasis because GR regulates the expression of biliary transport systems including the human BA transporters NTCP, ASBT, and OSTα/β (see Fig. 1).^216–218^ In addition, GR ligands may also modulate the function of other NRs including CAR, a primary GR response gene,^219^ as well as PXR and RXRα.^219,220^ On the other hand, GR activation promotes cholestasis in mice by repressing the beneficial transcriptional activity of FXR,^221^ although such potentially negative effects have never been reported clinically in cholestatic patients. Nevertheless, serum BA levels are elevated in patients with increased serum glucocorticoid levels, such as Cushing disease or obesity, in comparison with healthy individuals, and correlate with elevated glucocorticoid levels. This induction of BA levels by GR ligands can also be explained by recruiting corepressor complexes to FXR and thereby blocking its transcriptional activity.^221^

## GR as therapeutic target

Activation of GR by glucocorticoids is widely used to treat inflammatory and autoimmune diseases^222^ and have also been tested for treatment of various cholestatic disorders including PBC.^223^ Notably, in addition to their classic anti-inflammatory and immunomodulatory effects, GR ligands may also have anti-cholestatic effects through modulation of transporters. One of the most notable mechanisms of GR activation in chronic inflammatory bile duct disorders such as PBC may include stimulatory effects on AE2 expression, thus increasing cholangiocyte bicarbonate secretion^224,225^ and stimulation/restoration of the biliary bicarbonate umbrella (see Fig. 1). This effect is especially interesting in the context of reduced AE2 expression and function in the liver and inflammatory cells of PBC patients,^226,227^ which may be responsible for vulnerable cholangiocytes favoring an auto-immune hit on the bile ducts. Increased AE2 expression resulting in an increase of biliary bicarbonate secretion by UDCA and dexamethasone combination but not by UDCA or dexamethasone alone^225^ could provide a potential explanation for the observed beneficial effects of the combination of glucocorticoids and UDCA. Of note, UDCA also activates GR^228,229^ and promotes GR translocation in the nucleus in a ligand-independent manner,^230^ favoring a combination therapy of glucocorticoids and UDCA in PBC patients from a mechanistic point of view.

Although (combination) therapy with steroids may be clinically beneficial, their use is limited by classic side effects including bone loss,^231^ which outweigh the potential benefits. Moreover, it has been shown that patients receiving glucocorticoids have increased BA synthesis (see earlier discussion) and are prone to gallstone diseases.^232^ Use of glucocorticoids is considered an independent risk factor for cholelithiasis.^233^ Budesonide, a non-halogenated corticosteroid with a high GR-binding affinity and extensive hepatic first-pass metabolism-limiting (extrahepatic) side effects, may be an attractive alternative. Apart from GR-mediated effects, the induction of CYP3A4 via a PXR-dependent mechanism and thereby induction of BA detoxification, may also be an argument for the use of budesonide in inflammation-driven cholestatic diseases. Two randomized control trials have reported an additional benefit of budesonide and UDCA combination therapy on serum parameters of cholestasis and liver histology in PBC patients (stage I to III) in comparison to UDCA monotherapy.^234,235^ However, in a study focusing on a subgroup of patients who did not respond to UDCA monotherapy (including patients with end-stage disease), significant increases in Mayo Risk Score were reported, despite beneficial effects on bilirubin and alkaline phosphatase levels with additional budesonide treatment.^236^ The summary of reported data allows the conclusion that budesonide in combination with UDCA has favorable results on biochemical and histologic parameters in early-stage PBC, but not late-stage disease, where budesonide is contra-indicated (reports of severe side effects including portal vein thrombosis and death).^237^

## Ursodeoxycholic acid –– current anti-cholestatic drug standard and its effects on NRs

UDCA is currently used as a therapeutic standard in cholestasis and has multiple beneficial mechanisms,^238^ which may be mediated to at least in part by NRs. Although these various mechanisms of action of UDCA have been studied in detail in the last decades, the complete picture underlying the beneficial effects of UDCA remains to be determined. Notably, UDCA does not activate FXR^7,11,239^ and has low affinity to GR,^228^ but may activate PXR indirectly after its conversion to LCA by intestinal flora.^8,9^ In addition, UDCA induced expression of protective cathelicidin via activation of VDR in cultured biliary epithelial cells and induced both VDR and cathelicidin gene expression in livers of PBC patients.^82^ Furthermore, UDCA partially corrected calcium malabsorption in patients with PBC, who display low bone mass density and reduced fractional calcium malabsorption.^240^ Of note, UDCA may indirectly even counteract FXR activation by decreasing the relative concentrations of endogenous BA as more efficient FXR ligands. These examples indicate that direct or potentially indirect interactions with several NRs or transcriptional factors may be responsible for beneficial effects of UDCA. Importantly, several UDCA derivatives have been synthesized to potentiate the UDCA actions. As such, a 24-*nor*ursodeoxycholic acid (*nor*UDCA) showed beneficial effects in the Mdr2 knockout mouse model of biliary fibrosis.^241–243^ Anti-cholestatic, anti-fibrotic, and anti-inflammatory effects of *nor*UDCA were associated with induction of phase I and phase II detoxification enzymes with simultaneous induction of basolateral efflux systems, resulting in alternative renal BA excretion.^241,242^ In addition, *nor*UDCA induced induction of bicarbonate-rich bile flow. However, similar to its parent drug UDCA, no NR has been identified as a potential target for *nor*UDCA and generation of bicarbonate-rich bile flow by *nor*UDCA is thought to be mediated by the cholehepatic shunting of the compound.^242,244^ Although no NRs have been identified so far as a target for *nor*UDCA, a characteristic pattern of induction of CAR-regulated genes was observed in the gene expression array study, suggesting CAR involvement in the anti-cholestatic effect of this compound.^243^ Furthermore, *nor*UDCA has profound beneficial effects on lipoprotein composition, and hepatic lipid metabolism.^243,245^ These properties make *nor*UDCA a very attractive therapeutic candidate for cholestatic and metabolic liver diseases.

## Summary and future perspectives

NRs control several important hepatic functions involved in the pathophysiology of cholestatic liver disease such as BA homeostasis and enterohepatic circulation of BAs as well as hepatic inflammation and fibrosis. Novel concepts on NR (patho)physiology have successfully been integrated in the understanding of the development of cholestasis. At present, many drugs used as standard treatments for cholestasis act via NRs and stimulation of their target genes. A revolution of expanding use of NR targeting in the therapy for cholestatic diseases is being witnessed. The translation of expanding knowledge on NRs should result in optimizing the current standard therapy with careful selection of patients’ subgroups benefiting from such novel NR-directed approaches.

## References

[1] Erlinger S. (1985). What is cholestasis in 1985?. J Hepatol.

[2] Trauner M., Meier P.J., Boyer J.L. (1998). Molecular pathogenesis of cholestasis. N Engl J Med.

[3] Wagner M., Zollner G., Trauner M. (2011). Nuclear receptors in liver disease. Hepatology.

[4] Trauner M., Halilbasic E. (2011). Nuclear receptors as new perspective for the management of liver diseases. Gastroenterology.

[5] McKenna N.J., Lanz R.B., O'Malley B.W. (1999). Nuclear receptor coregulators: cellular and molecular biology. Endocr Rev.

[6] Perissi V., Rosenfeld M.G. (2005). Controlling nuclear receptors: the circular logic of cofactor cycles. Nat Rev Mol Cell Biol.

[7] Makishima M., Okamoto A.Y., Repa J.J. (1999). Identification of a nuclear receptor for bile acids. Science.

[8] Staudinger J.L., Goodwin B., Jones S.A. (2001). The nuclear receptor PXR is a lithocholic acid sensor that protects against liver toxicity. Proc Natl Acad Sci U S A.

[9] Xie W., Radominska-Pandya A., Shi Y. (2001). An essential role for nuclear receptors SXR/PXR in detoxification of cholestatic bile acids. Proc Natl Acad Sci U S A.

[10] Makishima M., Lu T.T., Xie W. (2002). Vitamin D receptor as an intestinal bile acid sensor. Science.

[11] Parks D.J., Blanchard S.G., Bledsoe R.K. (1999). Bile acids: natural ligands for an orphan nuclear receptor. Science.

[12] Wang H., Chen J., Hollister K. (1999). Endogenous bile acids are ligands for the nuclear receptor FXR/BAR. Mol Cell.

[13] Forman B.M., Goode E., Chen J. (1995). Identification of a nuclear receptor that is activated by farnesol metabolites. Cell.

[14] Lu T.T., Repa J.J., Mangelsdorf D.J. (2001). Orphan nuclear receptors as eLiXiRs and FiXeRs of sterol metabolism. J Biol Chem.

[15] Zhang Y., Kast-Woelbern H.R., Edwards P.A. (2003). Natural structural variants of the nuclear receptor farnesoid X receptor affect transcriptional activation. J Biol Chem.

[16] Seol W., Choi H.S., Moore D.D. (1995). Isolation of proteins that interact specifically with the retinoid X receptor: two novel orphan receptors. Mol Endocrinol.

[17] Laffitte B.A., Kast H.R., Nguyen C.M. (2000). Identification of the DNA binding specificity and potential target genes for the farnesoid X-activated receptor. J Biol Chem.

[18] Huber R.M., Murphy K., Miao B. (2002). Generation of multiple farnesoid-X-receptor isoforms through the use of alternative promoters. Gene.

[19] Downes M., Verdecia M.A., Roecker A.J. (2003). A chemical, genetic, and structural analysis of the nuclear bile acid receptor FXR. Mol Cell.

[20] Anisfeld A.M., Kast-Woelbern H.R., Meyer M.E. (2003). Syndecan-1 expression is regulated in an isoform specific manner by the farnesoid-X receptor. J Biol Chem.

[21] Denson L.A., Sturm E., Echevarria W. (2001). The orphan nuclear receptor, shp, mediates bile acid-induced inhibition of the rat bile acid transporter, ntcp. Gastroenterology.

[22] Ananthanarayanan M., Balasubramanian N., Makishima M. (2001). Human bile salt export pump promoter is transactivated by the farnesoid X receptor/bile acid receptor. J Biol Chem.

[23] Gerloff T., Geier A., Roots I. (2002). Functional analysis of the rat bile salt export pump gene promoter. Eur J Biochem.

[24] Plass J.R., Mol O., Heegsma J. (2002). Farnesoid X receptor and bile salts are involved in transcriptional regulation of the gene encoding the human bile salt export pump. Hepatology.

[25] Eloranta J.J., Kullak-Ublick G.A. (2005). Coordinate transcriptional regulation of bile acid homeostasis and drug metabolism. Arch Biochem Biophys.

[26] Gupta S., Pandak W.M., Hylemon P.B. (2002). LXR alpha is the dominant regulator of CYP7A1 transcription. Biochem Biophys Res Commun.

[27] Brendel C., Schoonjans K., Botrugno O.A. (2002). The small heterodimer partner interacts with the liver X receptor alpha and represses its transcriptional activity. Mol Endocrinol.

[28] Kir S., Zhang Y., Gerard R.D. (2012). Nuclear receptors HNF4-alpha and LRH-1 cooperate in regulating Cyp7a1 in Vivo. J Biol Chem.

[29] Abrahamsson A., Gustafsson U., Ellis E. (2005). Feedback regulation of bile acid synthesis in human liver: importance of HNF-4alpha for regulation of CYP7A1. Biochem Biophys Res Commun.

[30] Holt J.A., Luo G., Billin A.N. (2003). Definition of a novel growth factor-dependent signal cascade for the suppression of bile acid biosynthesis. Genes Dev.

[31] Inagaki T., Choi M., Moschetta A. (2005). Fibroblast growth factor 15 functions as an enterohepatic signal to regulate bile acid homeostasis. Cell Metab.

[32] Kim I., Ahn S.H., Inagaki T. (2007). Differential regulation of bile acid homeostasis by the farnesoid X receptor in liver and intestine. J Lipid Res.

[33] Choi M., Moschetta A., Bookout A.L. (2006). Identification of a hormonal basis for gallbladder filling. Nat Med.

[34] Kir S., Beddow S.A., Samuel V.T. (2011). FGF19 as a postprandial, insulin-independent activator of hepatic protein and glycogen synthesis. Science.

[35] Tomlinson E., Fu L., John L. (2002). Transgenic mice expressing human fibroblast growth factor-19 display increased metabolic rate and decreased adiposity. Endocrinology.

[36] Fu L., John L.M., Adams S.H. (2004). Fibroblast growth factor 19 increases metabolic rate and reverses dietary and leptin-deficient diabetes. Endocrinology.

[37] Schaap F.G., van der Gaag N.A., Gouma D.J. (2009). High expression of the bile salt-homeostatic hormone fibroblast growth factor 19 in the liver of patients with extrahepatic cholestasis. Hepatology.

[38] Zweers S.J., Booij K.A., Komuta M. (2012). The human gallbladder secretes fibroblast growth factor 19 into bile: towards defining the role of fibroblast growth factor 19 in the enterobiliary tract. Hepatology.

[39] Xu Y., Watanabe T., Tanigawa T. (2010). Bile acids induce cdx2 expression through the farnesoid x receptor in gastric epithelial cells. J Clin Biochem Nutr.

[40] Dawson P.A., Hubbert M., Haywood J. (2005). The heteromeric organic solute transporter alpha-beta, Ostalpha-Ostbeta, is an ileal basolateral bile acid transporter. J Biol Chem.

[41] Boyer J.L., Trauner M., Mennone A. (2006). Upregulation of a basolateral FXR-dependent bile acid efflux transporter OSTalpha-OSTbeta in cholestasis in humans and rodents. Am J Physiol Gastrointest Liver Physiol.

[42] Zollner G., Marschall H.U., Wagner M. (2006). Role of nuclear receptors in the adaptive response to bile acids and cholestasis: pathogenetic and therapeutic considerations. Mol Pharm.

[43] Huang L., Zhao A., Lew J.L. (2003). Farnesoid X-receptor activates transcription of the phospholipid pump MDR3. J Biol Chem.

[44] Kast H.R., Goodwin B., Tarr P.T. (2002). Regulation of multidrug resistance-associated protein 2 (ABCC2) by the nuclear receptors pregnane X receptor, farnesoid X-activated receptor, and constitutive androstane receptor. J Biol Chem.

[45] Deng R., Yang D., Yang J. (2006). Oxysterol 22(R)-hydroxycholesterol induces the expression of the bile salt export pump through nuclear receptor farsenoid X receptor but not liver X receptor. J Pharmacol Exp Ther.

[46] Wang S., Lai K., Moy F.J. (2006). The nuclear hormone receptor farnesoid X receptor (FXR) is activated by androsterone. Endocrinology.

[47] Carter B.A., Prendergast D.R., Taylor O.A. (2007). Stigmasterol, a soy lipid-derived phytosterol, is an antagonist of the bile acid nuclear receptor FXR. Pediatr Res.

[48] Van Mil S.W., Milona A., Dixon P.H. (2007). Functional variants of the central bile acid sensor FXR identified in intrahepatic cholestasis of pregnancy. Gastroenterology.

[49] Alvarez L., Jara P., Sanchez-Sabate E. (2004). Reduced hepatic expression of farnesoid X receptor in hereditary cholestasis associated to mutation in ATP8B1. Hum Mol Genet.

[50] Chen F., Ananthanarayanan M., Emre S. (2004). Progressive familial intrahepatic cholestasis, type 1, is associated with decreased farnesoid X receptor activity. Gastroenterology.

[51] Pauli-Magnus C., Meier P.J. (2006). Hepatobiliary transporters and drug-induced cholestasis. Hepatology.

[52] Marzolini C., Tirona R.G., Gervasini G. (2007). A common polymorphism in the bile acid receptor farnesoid x receptor is associated with decreased hepatic target gene expression. Mol Endocrinol.

[53] Ismair M.G., Stieger B., Cattori V. (2001). Hepatic uptake of cholecystokinin octapeptide by organic anion-transporting polypeptides OATP4 and OATP8 of rat and human liver. Gastroenterology.

[54] Kullak-Ublick G.A., Ismair M.G., Stieger B. (2001). Organic anion-transporting polypeptide B (OATP-B) and its functional comparison with three other OATPs of human liver. Gastroenterology.

[55] Moschetta A., Bookout A.L., Mangelsdorf D.J. (2004). Prevention of cholesterol gallstone disease by FXR agonists in a mouse model. Nat Med.

[56] Kovacs P., Kress R., Rocha J. (2008). Variation of the gene encoding the nuclear bile salt receptor FXR and gallstone susceptibility in mice and humans. J Hepatol.

[57] Bertolotti M., Gabbi C., Anzivino C. (2006). Decreased hepatic expression of PPAR-gamma coactivator-1 in cholesterol cholelithiasis. Eur J Clin Invest.

[58] Kanaya E., Shiraki T., Jingami H. (2004). The nuclear bile acid receptor FXR is activated by PGC-1alpha in a ligand-dependent manner. Biochem J.

[59] Zhang Y., Castellani L.W., Sinal C.J. (2004). Peroxisome proliferator-activated receptor-gamma coactivator 1alpha (PGC-1alpha) regulates triglyceride metabolism by activation of the nuclear receptor FXR. Genes Dev.

[60] Zollner G., Fickert P., Silbert D. (2003). Adaptive changes in hepatobiliary transporter expression in primary biliary cirrhosis. J Hepatol.

[61] Zollner G., Wagner M., Moustafa T. (2006). Coordinated induction of bile acid detoxification and alternative elimination in mice: role of FXR-regulated organic solute transporter-alpha/beta in the adaptive response to bile acids. Am J Physiol Gastrointest Liver Physiol.

[62] Donner M.G., Keppler D. (2001). Up-regulation of basolateral multidrug resistance protein 3 (Mrp3) in cholestatic rat liver. Hepatology.

[63] Shoda J., Kano M., Oda K. (2001). The expression levels of plasma membrane transporters in the cholestatic liver of patients undergoing biliary drainage and their association with the impairment of biliary secretory function. Am J Gastroenterol.

[64] Ogawa K., Suzuki H., Hirohashi T. (2000). Characterization of inducible nature of MRP3 in rat liver. Am J Physiol Gastrointest Liver Physiol.

[65] Keitel V., Burdelski M., Warskulat U. (2005). Expression and localization of hepatobiliary transport proteins in progressive familial intrahepatic cholestasis. Hepatology.

[66] Zollner G., Wagner M., Fickert P. (2007). Expression of bile acid synthesis and detoxification enzymes and the alternative bile acid efflux pump MRP4 in patients with primary biliary cirrhosis. Liver Int.

[67] Kim I., Morimura K., Shah Y. (2007). Spontaneous hepatocarcinogenesis in farnesoid X receptor-null mice. Carcinogenesis.

[68] Yang F., Huang X., Yi T. (2007). Spontaneous development of liver tumors in the absence of the bile acid receptor farnesoid X receptor. Cancer Res.

[69] Zhang Y., Xu P., Park K. (2008). Orphan receptor small heterodimer partner suppresses tumorigenesis by modulating cyclin D1 expression and cellular proliferation. Hepatology.

[70] He N., Park K., Zhang Y. (2008). Epigenetic inhibition of nuclear receptor small heterodimer partner is associated with and regulates hepatocellular carcinoma growth. Gastroenterology.

[71] Knisely A.S., Strautnieks S.S., Meier Y. (2006). Hepatocellular carcinoma in ten children under five years of age with bile salt export pump deficiency. Hepatology.

[72] Zhang Y., Soto J., Park K. (2010). Nuclear receptor SHP, a death receptor that targets mitochondria, induces apoptosis and inhibits tumor growth. Mol Cell Biol.

[73] Chen W.D., Wang Y.D., Zhang L. (2010). Farnesoid X receptor alleviates age-related proliferation defects in regenerating mouse livers by activating forkhead box m1b transcription. Hepatology.

[74] De Gottardi A., Touri F., Maurer C.A. (2004). The bile acid nuclear receptor FXR and the bile acid binding protein IBABP are differently expressed in colon cancer. Dig Dis Sci.

[75] Journe F., Durbecq V., Chaboteaux C. (2009). Association between farnesoid X receptor expression and cell proliferation in estrogen receptor-positive luminal-like breast cancer from postmenopausal patients. Breast Cancer Res Treat.

[76] Liu Y., Binz J., Numerick M.J. (2003). Hepatoprotection by the farnesoid X receptor agonist GW4064 in rat models of intra- and extrahepatic cholestasis. J Clin Invest.

[77] Fiorucci S., Clerici C., Antonelli E. (2005). Protective effects of 6-ethyl chenodeoxycholic acid, a farnesoid X receptor ligand, in estrogen-induced cholestasis. J Pharmacol Exp Ther.

[78] Baghdasaryan A., Claudel T., Gumhold J. (2011). Dual FXR/TGR5 agonist INT-767 reduces liver injury in the Mdr2−/−(Abcb4−/−) mouse cholangiopathy model by promoting biliary HCO3− output. Hepatology.

[79] McMurtrie H.L., Cleary H.J., Alvarez B.V. (2004). The bicarbonate transport metabolon. J Enzyme Inhib Med Chem.

[80] Chignard N., Mergey M., Barbu V. (2005). VPAC1 expression is regulated by FXR agonists in the human gallbladder epithelium. Hepatology.

[81] Cho W.K., Boyer J.L. (1999). Vasoactive intestinal polypeptide is a potent regulator of bile secretion from rat cholangiocytes. Gastroenterology.

[82] D'Aldebert E., Biyeyeme Bi Mve M.J., Mergey M. (2009). Bile salts control the antimicrobial peptide cathelicidin through nuclear receptors in the human biliary epithelium. Gastroenterology.

[83] Wang Y.D., Chen W.D., Wang M. (2008). Farnesoid X receptor antagonizes nuclear factor kappaB in hepatic inflammatory response. Hepatology.

[84] Li Y.T., Swales K.E., Thomas G.J. (2007). Farnesoid X receptor ligands inhibit vascular smooth muscle cell inflammation and migration. Arterioscler Thromb Vasc Biol.

[85] Xu Z., Huang G., Gong W. (2012). FXR ligands protect against hepatocellular inflammation via SOCS3 induction. Cell Signal.

[86] Gadaleta R.M., van Erpecum K.J., Oldenburg B. (2011). Farnesoid X receptor activation inhibits inflammation and preserves the intestinal barrier in inflammatory bowel disease. Gut.

[87] Ding J.W., Andersson R., Soltesz V. (1993). The role of bile and bile acids in bacterial translocation in obstructive jaundice in rats. Eur Surg Res.

[88] Lorenzo-Zuniga V., Bartoli R., Planas R. (2003). Oral bile acids reduce bacterial overgrowth, bacterial translocation, and endotoxemia in cirrhotic rats. Hepatology.

[89] Cahill C.J. (1983). Prevention of postoperative renal failure in patients with obstructive jaundice–the role of bile salts. Br J Surg.

[90] Inagaki T., Moschetta A., Lee Y.K. (2006). Regulation of antibacterial defense in the small intestine by the nuclear bile acid receptor. Proc Natl Acad Sci U S A.

[91] Modica S., Petruzzelli M., Bellafante E. (2012). Selective activation of nuclear bile acid receptor FXR in the intestine protects mice against cholestasis. Gastroenterology.

[92] Fiorucci S., Antonelli E., Rizzo G. (2004). The nuclear receptor SHP mediates inhibition of hepatic stellate cells by FXR and protects against liver fibrosis. Gastroenterology.

[93] Fiorucci S., Rizzo G., Antonelli E. (2005). A FXR-SHP regulatory cascade modulates TIMP-1 and MMPs expression in HSCs and promotes resolution of liver fibrosis. J Pharmacol Exp Ther.

[94] Fickert P., Fuchsbichler A., Wagner M. (2009). The role of the hepatocyte cytokeratin network in bile formation and resistance to bile acid challenge and cholestasis in mice. Hepatology.

[95] Mason A., Luketic V., Lindor K. (2010). Farnesoid-X Receptor agonists: a new class of drugs for the treatment of PBC? An international study evaluating the addition of obeticholic acid (INT-747) to ursodeoxycholic acid. Hepatology.

[96] Hirschfield G., Mason A., Gordon S. (2011). A long term safty extenion trial of the farnesoid X receptor (FXR) agonist obeticholic acid (OCA) and UDCA in pimary biliary cirrhosis (PBC). Hepatology.

[97] Kowdley K.V., Jones D., Luketic V., The OCA PBC Study Group (2012). An international study evaluating the farnesoid X receptor agonist obeticholic acid as monotherapy in PBC. J Hepatol.

[98] Kliewer S.A., Goodwin B., Willson T.M. (2002). The nuclear pregnane X receptor: a key regulator of xenobiotic metabolism. Endocr Rev.

[99] Blumberg B., Sabbagh W., Juguilon H. (1998). SXR, a novel steroid and xenobiotic-sensing nuclear receptor. Genes Dev.

[100] Kliewer S.A., Moore J.T., Wade L. (1998). An orphan nuclear receptor activated by pregnanes defines a novel steroid signaling pathway. Cell.

[101] Lehmann J.M., McKee D.D., Watson M.A. (1998). The human orphan nuclear receptor PXR is activated by compounds that regulate CYP3A4 gene expression and cause drug interactions. J Clin Invest.

[102] Moore L.B., Parks D.J., Jones S.A. (2000). Orphan nuclear receptors constitutive androstane receptor and pregnane X receptor share xenobiotic and steroid ligands. J Biol Chem.

[103] Wentworth J.M., Agostini M., Love J. (2000). St John's wort, a herbal antidepressant, activates the steroid X receptor. J Endocrinol.

[104] Huang W., Zhang J., Chua S.S. (2003). Induction of bilirubin clearance by the constitutive androstane receptor (CAR). Proc Natl Acad Sci U S A.

[105] Wada T., Gao J., Xie W. (2009). PXR and CAR in energy metabolism. Trends Endocrinol Metab.

[106] Xie W., Barwick J.L., Simon C.M. (2000). Reciprocal activation of xenobiotic response genes by nuclear receptors SXR/PXR and CAR. Genes Dev.

[107] Jung D., Mangelsdorf D.J., Meyer U.A. (2006). Pregnane X receptor is a target of farnesoid X receptor. J Biol Chem.

[108] Castano G., Burgueno A., Fernandez Gianotti T. (2010). The influence of common gene variants of the xenobiotic receptor (PXR) in genetic susceptibility to intrahepatic cholestasis of pregnancy. Aliment Pharmacol Ther.

[109] Owen B.M., Van Mil S.W., Boudjelal M. (2008). Sequencing and functional assessment of hPXR (NR1I2) variants in intrahepatic cholestasis of pregnancy. Xenobiotica.

[110] Karlsen T.H., Lie B.A., Frey Froslie K. (2006). Polymorphisms in the steroid and xenobiotic receptor gene influence survival in primary sclerosing cholangitis. Gastroenterology.

[111] Chai J., He Y., Cai S.Y. (2012). Elevated hepatic multidrug resistance-associated protein 3/ATP-binding cassette subfamily C 3 expression in human obstructive cholestasis is mediated through tumor necrosis factor alpha and c-Jun NH2-terminal kinase/stress-activated protein kinase-signaling pathway. Hepatology.

[112] Chai J., Luo D., Wu X. (2011). Changes of organic anion transporter MRP4 and related nuclear receptors in human obstructive cholestasis. J Gastrointest Surg.

[113] Chen H.L., Liu Y.J., Wu S.H. (2008). Expression of hepatocyte transporters and nuclear receptors in children with early and late-stage biliary atresia. Pediatr Res.

[114] Hanada K., Nakai K., Tanaka H. (2012). Effect of nuclear receptor downregulation on hepatic expression of cytochrome P450 and transporters in chronic hepatitis C in association with fibrosis development. Drug Metab Pharmacokinet.

[115] Bachs L., Pares A., Elena M. (1989). Comparison of rifampicin with phenobarbitone for treatment of pruritus in biliary cirrhosis. Lancet.

[116] Cancado E.L., Leitao R.M., Carrilho F.J. (1998). Unexpected clinical remission of cholestasis after rifampicin therapy in patients with normal or slightly increased levels of gamma-glutamyl transpeptidase. Am J Gastroenterol.

[117] Yerushalmi B., Sokol R.J., Narkewicz M.R. (1999). Use of rifampin for severe pruritus in children with chronic cholestasis. J Pediatr Gastroenterol Nutr.

[118] Marschall H.U., Wagner M., Zollner G. (2005). Complementary stimulation of hepatobiliary transport and detoxification systems by rifampicin and ursodeoxycholic acid in humans. Gastroenterology.

[119] Kremer A.E., Martens J.J., Kulik W. (2010). Lysophosphatidic acid is a potential mediator of cholestatic pruritus. Gastroenterology.

[120] Kremer A.E., van Dijk R., Leckie P. (2012). Serum autotaxin is increased in pruritus of cholestasis, but not of other origin, and responds to therapeutic interventions. Hepatology.

[121] Stiehl A., Thaler M.M., Admirand W.H. (1972). The effects of phenobarbital on bile salts and bilirubin in patients with intrahepatic and extrahepatic cholestasis. N Engl J Med.

[122] Bloomer J.R., Boyer J.L. (1975). Phenobarbital effects in cholestatic liver diseases. Ann Intern Med.

[123] Huang W., Zhang J., Moore D.D. (2004). A traditional herbal medicine enhances bilirubin clearance by activating the nuclear receptor CAR. J Clin Invest.

[124] Chen C., Staudinger J.L., Klaassen C.D. (2003). Nuclear receptor, pregname X receptor, is required for induction of UDP-glucuronosyltranferases in mouse liver by pregnenolone-16 alpha-carbonitrile. Drug Metab Dispos.

[125] Teng S., Piquette-Miller M. (2007). Hepatoprotective role of PXR activation and MRP3 in cholic acid-induced cholestasis. Br J Pharmacol.

[126] Beilke L.D., Aleksunes L., Holland R. (2009). Car-mediated changes in bile acid composition contributes to hepatoprotection from lca-induced liver injury in mice. Drug Metab Dispos.

[127] Wagner M., Halilbasic E., Marschall H.U. (2005). CAR and PXR agonists stimulate hepatic bile acid and bilirubin detoxification and elimination pathways in mice. Hepatology.

[128] Renga B., Migliorati M., Mencarelli A. (2011). Farnesoid X receptor suppresses constitutive androstane receptor activity at the multidrug resistance protein-4 promoter. Biochim Biophys Acta.

[129] Haughton E.L., Tucker S.J., Marek C.J. (2006). Pregnane X receptor activators inhibit human hepatic stellate cell transdifferentiation in vitro. Gastroenterology.

[130] Marek C.J., Tucker S.J., Konstantinou D.K. (2005). Pregnenolone-16alpha-carbonitrile inhibits rodent liver fibrogenesis via PXR (pregnane X receptor)-dependent and PXR-independent mechanisms. Biochem J.

[131] Wallace K., Cowie D.E., Konstantinou D.K. (2010). The PXR is a drug target for chronic inflammatory liver disease. J Steroid Biochem Mol Biol.

[132] Zhou C., Tabb M.M., Nelson E.L. (2006). Mutual repression between steroid and xenobiotic receptor and NF-kappaB signaling pathways links xenobiotic metabolism and inflammation. J Clin Invest.

[133] Paunescu E. (1970). In vivo and in vitro suppression of humoral and cellular immunological response by rifampicin. Nature.

[134] Hu G., Xu C., Staudinger J.L. (2010). Pregnane X receptor is SUMOylated to repress the inflammatory response. J Pharmacol Exp Ther.

[135] Dai G., He L., Bu P. (2008). Pregnane X receptor is essential for normal progression of liver regeneration. Hepatology.

[136] Columbano A., Ledda-Columbano G.M., Pibiri M. (2005). Gadd45beta is induced through a CAR-dependent, TNF-independent pathway in murine liver hyperplasia. Hepatology.

[137] Huang W., Zhang J., Washington M. (2005). Xenobiotic stress induces hepatomegaly and liver tumors via the nuclear receptor constitutive androstane receptor. Mol Endocrinol.

[138] Yamamoto Y., Moore R., Goldsworthy T.L. (2004). The orphan nuclear receptor constitutive active/androstane receptor is essential for liver tumor promotion by phenobarbital in mice. Cancer Res.

[139] Campbell M.J., Adorini L. (2006). The vitamin D receptor as a therapeutic target. Expert Opin Ther Targets.

[140] Gascon-Barre M., Demers C., Mirshahi A. (2003). The normal liver harbors the vitamin D nuclear receptor in nonparenchymal and biliary epithelial cells. Hepatology.

[141] Abramovitch S., Dahan-Bachar L., Sharvit E. (2011). Vitamin D inhibits proliferation and profibrotic marker expression in hepatic stellate cells and decreases thioacetamide-induced liver fibrosis in rats. Gut.

[142] Chen X., Chen F., Liu S. (2006). Transactivation of rat apical sodium-dependent bile acid transporter and increased bile acid transport by 1alpha, 25-dihydroxyvitamin D3 via the vitamin D receptor. Mol Pharmacol.

[143] McCarthy T.C., Li X., Sinal C.J. (2005). Vitamin D receptor-dependent regulation of colon multidrug resistance-associated protein 3 gene expression by bile acids. J Biol Chem.

[144] Echchgadda I., Song C.S., Roy A.K. (2004). Dehydroepiandrosterone sulfotransferase is a target for transcriptional induction by the vitamin D receptor. Mol Pharmacol.

[145] Chatterjee B., Echchgadda I., Song C.S. (2005). Vitamin D receptor regulation of the steroid/bile acid sulfotransferase SULT2A1. Methods Enzymol.

[146] Honjo Y., Sasaki S., Kobayashi Y. (2006). 1,25-dihydroxyvitamin D3 and its receptor inhibit the chenodeoxycholic acid-dependent transactivation by farnesoid X receptor. J Endocrinol.

[147] Vogel A., Strassburg C.P., Manns M.P. (2002). Genetic association of vitamin D receptor polymorphisms with primary biliary cirrhosis and autoimmune hepatitis. Hepatology.

[148] Tanaka A., Nezu S., Uegaki S. (2009). Vitamin D receptor polymorphisms are associated with increased susceptibility to primary biliary cirrhosis in Japanese and Italian populations. J Hepatol.

[149] Fan L., Tu X., Zhu Y. (2005). Genetic association of vitamin D receptor polymorphisms with autoimmune hepatitis and primary biliary cirrhosis in the Chinese. J Gastroenterol Hepatol.

[150] Halmos B., Szalay F., Cserniczky T. (2000). Association of primary biliary cirrhosis with vitamin D receptor BsmI genotype polymorphism in a Hungarian population. Dig Dis Sci.

[151] Kempinska-Podhorecka A., Wunsch E., Jarowicz T. (2012). Vitamin D receptor polymorphisms predispose to primary biliary cirrhosis and severity of the disease in polish population. Gastroenterol Res Pract.

[152] Wikstrom Shemer E., Marschall H.U. (2010). Decreased 1,25-dihydroxy vitamin D levels in women with intrahepatic cholestasis of pregnancy. Acta Obstet Gynecol Scand.

[153] Barchetta I., Carotti S., Labbadia G. (2012). Liver VDR, CYP2R1 and CYP27A1 expression: relationship with liver histology and vitamin D3 levels in patients with NASH or HCV hepatitis. Hepatology.

[154] Spina C.S., Ton L., Yao M. (2007). Selective vitamin D receptor modulators and their effects on colorectal tumor growth. J Steroid Biochem Mol Biol.

[155] de Lyra E.C., da Silva I.A., Katayama M.L. (2006). 25(OH)D3 and 1,25(OH)2D3 serum concentration and breast tissue expression of 1alpha-hydroxylase, 24-hydroxylase and Vitamin D receptor in women with and without breast cancer. J Steroid Biochem Mol Biol.

[156] Li Q., Gao Y., Jia Z. (2012). Dysregulated Kruppel-like factor 4 and vitamin D receptor signaling contribute to progression of hepatocellular carcinoma. Gastroenterology.

[157] Ruiz-Gaspa S., Guanabens N., Enjuanes A. (2010). Lithocholic acid downregulates vitamin D effects in human osteoblasts. Eur J Clin Invest.

[158] Al-Harthy N., Kumagi T., Coltescu C. (2010). The specificity of fatigue in primary biliary cirrhosis: evaluation of a large clinic practice. Hepatology.

[159] Ogura M., Nishida S., Ishizawa M. (2009). Vitamin D3 modulates the expression of bile acid regulatory genes and represses inflammation in bile duct-ligated mice. J Pharmacol Exp Ther.

[160] Chawla A., Repa J.J., Evans R.M. (2001). Nuclear receptors and lipid physiology: opening the X-files. Science.

[161] Krey G., Braissant O., L'Horset F. (1997). Fatty acids, eicosanoids, and hypolipidemic agents identified as ligands of peroxisome proliferator-activated receptors by coactivator-dependent receptor ligand assay. Mol Endocrinol.

[162] Kota B.P., Huang T.H., Roufogalis B.D. (2005). An overview on biological mechanisms of PPARs. Pharmacol Res.

[163] Bookout A.L., Jeong Y., Downes M. (2006). Anatomical profiling of nuclear receptor expression reveals a hierarchical transcriptional network. Cell.

[164] Braissant O., Foufelle F., Scotto C. (1996). Differential expression of peroxisome proliferator-activated receptors (PPARs): tissue distribution of PPAR-alpha, -beta, and -gamma in the adult rat. Endocrinology.

[165] Kliewer S.A., Xu H.E., Lambert M.H. (2001). Peroxisome proliferator-activated receptors: from genes to physiology. Recent Prog Horm Res.

[166] Tontonoz P., Hu E., Spiegelman B.M. (1994). Stimulation of adipogenesis in fibroblasts by PPAR gamma 2, a lipid-activated transcription factor. Cell.

[167] Fang H.L., Strom S.C., Cai H. (2005). Regulation of human hepatic hydroxysteroid sulfotransferase gene expression by the peroxisome proliferator-activated receptor alpha transcription factor. Mol Pharmacol.

[168] Barbier O., Duran-Sandoval D., Pineda-Torra I. (2003). Peroxisome proliferator-activated receptor alpha induces hepatic expression of the human bile acid glucuronidating UDP-glucuronosyltransferase 2B4 enzyme. J Biol Chem.

[169] Jung D., Fried M., Kullak-Ublick G.A. (2002). Human apical sodium-dependent bile salt transporter gene (SLC10A2) is regulated by the peroxisome proliferator-activated receptor alpha. J Biol Chem.

[170] Barbier O., Trottier J., Kaeding J. (2009). Lipid-activated transcription factors control bile acid glucuronidation. Mol Cell Biochem.

[171] Marrapodi M., Chiang J.Y. (2000). Peroxisome proliferator-activated receptor alpha (PPARalpha) and agonist inhibit cholesterol 7alpha-hydroxylase gene (CYP7A1) transcription. J Lipid Res.

[172] Patel D.D., Knight B.L., Soutar A.K. (2000). The effect of peroxisome-proliferator-activated receptor-alpha on the activity of the cholesterol 7 alpha-hydroxylase gene. Biochem J.

[173] Post S.M., Duez H., Gervois P.P. (2001). Fibrates suppress bile acid synthesis via peroxisome proliferator-activated receptor-alpha-mediated downregulation of cholesterol 7alpha-hydroxylase and sterol 27-hydroxylase expression. Arterioscler Thromb Vasc Biol.

[174] Roglans N., Vazquez-Carrera M., Alegret M. (2004). Fibrates modify the expression of key factors involved in bile-acid synthesis and biliary-lipid secretion in gallstone patients. Eur J Clin Pharmacol.

[175] Ghonem N., Ananthanarayanan M., Soroka C.J. (2012). Fenofibrate, a specific peroxisome proliferator-activated receptor alpha (PPARα) agonist, up-regulates MDR3/ABCB4 expression in human hepatocytes. Hepatology.

[176] Ghose R., Mulder J., von Furstenberg R.J. (2007). Rosiglitazone attenuates suppression of RXRalpha-dependent gene expression in inflamed liver. J Hepatol.

[177] Harada K., Nakanuma Y. (2010). Biliary innate immunity: function and modulation. Mediators Inflamm.

[178] Zhang F., Lu Y., Zheng S. (2012). Peroxisome proliferator-activated receptor-gamma cross-regulation of signaling events implicated in liver fibrogenesis. Cell Signal.

[179] Harada K., Isse K., Kamihira T. (2005). Th1 cytokine-induced downregulation of PPARgamma in human biliary cells relates to cholangitis in primary biliary cirrhosis. Hepatology.

[180] Miyahara T., Schrum L., Rippe R. (2000). Peroxisome proliferator-activated receptors and hepatic stellate cell activation. J Biol Chem.

[181] Matsumoto T., Miyazaki H., Nakahashi Y. (2004). Multidrug resistance3 is in situ detected in the liver of patients with primary biliary cirrhosis, and induced in human hepatoma cells by bezafibrate. Hepatol Res.

[182] Shoda J., Inada Y., Tsuji A. (2004). Bezafibrate stimulates canalicular localization of NBD-labeled PC in HepG2 cells by PPARalpha-mediated redistribution of ABCB4. J Lipid Res.

[183] Nakamuta M., Fujino T., Yada R. (2010). Therapeutic effect of bezafibrate against biliary damage: a study of phospholipid secretion via the PPARalpha-MDR3 pathway. Int J Clin Pharmacol Ther.

[184] Honda A., Ikegami T., Nakamuta M. (2012). Anticholestatic effects of bezafibrate in patients with primary biliary cirrhosis treated with ursodeoxycholic acid. Hepatology.

[185] Ohmoto K., Mitsui Y., Yamamoto S. (2001). Effect of bezafibrate in primary biliary cirrhosis: a pilot study. Liver.

[186] Yano K., Kato H., Morita S. (2002). Is bezafibrate histologically effective for primary biliary cirrhosis?. Am J Gastroenterol.

[187] Kanda T., Yokosuka O., Imazeki F. (2003). Bezafibrate treatment: a new medical approach for PBC patients?. J Gastroenterol.

[188] Kurihara T., Maeda A., Shigemoto M. (2002). Investigation into the efficacy of bezafibrate against primary biliary cirrhosis, with histological references from cases receiving long term monotherapy. Am J Gastroenterol.

[189] Kurihara T., Niimi A., Maeda A. (2000). Bezafibrate in the treatment of primary biliary cirrhosis: comparison with ursodeoxycholic acid. Am J Gastroenterol.

[190] Nakai S., Masaki T., Kurokohchi K. (2000). Combination therapy of bezafibrate and ursodeoxycholic acid in primary biliary cirrhosis: a preliminary study. Am J Gastroenterol.

[191] Han X.F., Wang Q.X., Liu Y. (2012). Efficacy of fenofibrate in Chinese patients with primary biliary cirrhosis partially responding to ursodeoxycholic acid therapy. J Dig Dis.

[192] Liberopoulos E.N., Florentin M., Elisaf M.S. (2010). Fenofibrate in primary biliary cirrhosis: a pilot study. Open Cardiovasc Med J.

[193] Levy C., Peter J.A., Nelson D.R. (2011). Pilot study: fenofibrate for patients with primary biliary cirrhosis and an incomplete response to ursodeoxycholic acid. Aliment Pharmacol Ther.

[194] Dohmen K., Mizuta T., Nakamuta M. (2004). Fenofibrate for patients with asymptomatic primary biliary cirrhosis. World J Gastroenterol.

[195] Itakura J., Izumi N., Nishimura Y. (2004). Prospective randomized crossover trial of combination therapy with bezafibrate and UDCA for primary biliary cirrhosis. Hepatol Res.

[196] Miyaguchi S., Ebinuma H., Imaeda H. (2000). A novel treatment for refractory primary biliary cirrhosis?. Hepatogastroenterology.

[197] Hazzan R., Tur-Kaspa R. (2010). Bezafibrate treatment of primary biliary cirrhosis following incomplete response to ursodeoxycholic acid. J Clin Gastroenterol.

[198] Iwasaki S., Akisawa N., Saibara T. (2007). Fibrate for treatment of primary biliary cirrhosis. Hepatol Res.

[199] Takeuchi Y., Ikeda F., Fujioka S. (2011). Additive improvement induced by bezafibrate in patients with primary biliary cirrhosis showing refractory response to ursodeoxycholic acid. J Gastroenterol Hepatol.

[200] Akbar S.M., Furukawa S., Nakanishi S. (2005). Therapeutic efficacy of decreased nitrite production by bezafibrate in patients with primary biliary cirrhosis. J Gastroenterol.

[201] Caroli-Bosc F.X., Le Gall P., Pugliese P. (2001). Role of fibrates and HMG-CoA reductase inhibitors in gallstone formation: epidemiological study in an unselected population. Dig Dis Sci.

[202] Hidaka M., Iwasaki S., Matsui T. (2010). Efficacy of bezafibrate for chronic GVHD of the liver after allogeneic hematopoietic stem cell transplantation. Bone Marrow Transplant.

[203] Landrier J.F., Thomas C., Grober J. (2004). Statin induction of liver fatty acid-binding protein (L-FABP) gene expression is peroxisome proliferator-activated receptor-alpha-dependent. J Biol Chem.

[204] Carrella M., Feldman D., Cogoi S. (1999). Enhancement of mdr2 gene transcription mediates the biliary transfer of phosphatidylcholine supplied by an increased biosynthesis in the pravastatin-treated rat. Hepatology.

[205] Hooiveld G.J., Vos T.A., Scheffer G.L. (1999). 3-Hydroxy-3-methylglutaryl-coenzyme A reductase inhibitors (statins) induce hepatic expression of the phospholipid translocase mdr2 in rats. Gastroenterology.

[206] Kurihara T., Akimoto M., Abe K. (1993). Experimental use of pravastatin in patients with primary biliary cirrhosis associated with hypercholesterolemia. Clin Ther.

[207] Kamisako T., Adachi Y. (1995). Marked improvement in cholestasis and hypercholesterolemia with simvastatin in a patient with primary biliary cirrhosis. Am J Gastroenterol.

[208] Ritzel U., Leonhardt U., Nather M. (2002). Simvastatin in primary biliary cirrhosis: effects on serum lipids and distinct disease markers. J Hepatol.

[209] Stojakovic T., Putz-Bankuti C., Fauler G. (2007). Atorvastatin in patients with primary biliary cirrhosis and incomplete biochemical response to ursodeoxycholic acid. Hepatology.

[210] Marra F., DeFranco R., Robino G. (2005). Thiazolidinedione treatment inhibits bile duct proliferation and fibrosis in a rat model of chronic cholestasis. World J Gastroenterol.

[211] Snow K.L., Moseley R.H. (2007). Effect of thiazolidinediones on bile acid transport in rat liver. Life Sci.

[212] Funk C., Ponelle C., Scheuermann G. (2001). Cholestatic potential of troglitazone as a possible factor contributing to troglitazone-induced hepatotoxicity: in vivo and in vitro interaction at the canalicular bile salt export pump (Bsep) in the rat. Mol Pharmacol.

[213] Baghdasaryan A., Claudel T., Kosters A. (2010). Curcumin improves sclerosing cholangitis in Mdr2−/− mice by inhibition of cholangiocyte inflammatory response and portal myofibroblast proliferation. Gut.

[214] Rivera-Espinoza Y., Muriel P. (2009). Pharmacological actions of curcumin in liver diseases or damage. Liver Int.

[215] Rose A.J., Vegiopoulos A., Herzig S. (2010). Role of glucocorticoids and the glucocorticoid receptor in metabolism: insights from genetic manipulations. J Steroid Biochem Mol Biol.

[216] Jung D., Fantin A.C., Scheurer U. (2004). Human ileal bile acid transporter gene ASBT (SLC10A2) is transactivated by the glucocorticoid receptor. Gut.

[217] Eloranta J.J., Jung D., Kullak-Ublick G.A. (2006). The human Na+-taurocholate cotransporting polypeptide gene is activated by glucocorticoid receptor and peroxisome proliferator-activated receptor-gamma coactivator-1alpha, and suppressed by bile acids via a small heterodimer partner-dependent mechanism. Mol Endocrinol.

[218] Khan A.A., Chow E.C., Porte R.J. (2009). Expression and regulation of the bile acid transporter, OSTalpha-OSTbeta in rat and human intestine and liver. Biopharm Drug Dispos.

[219] Pascussi J.M., Gerbal-Chaloin S., Fabre J.M. (2000). Dexamethasone enhances constitutive androstane receptor expression in human hepatocytes: consequences on cytochrome P450 gene regulation. Mol Pharmacol.

[220] Pascussi J.M., Gerbal-Chaloin S., Drocourt L. (2003). The expression of CYP2B6, CYP2C9 and CYP3A4 genes: a tangle of networks of nuclear and steroid receptors. Biochim Biophys Acta.

[221] Lu Y., Zhang Z., Xiong X. (2012). Glucocorticoids promote hepatic cholestasis in mice by inhibiting the transcriptional activity of the farnesoid x receptor. Gastroenterology.

[222] Gossard A.A., Lindor K.D. (2012). Autoimmune hepatitis: a review. J Gastroenterol.

[223] Poupon R. (2011). Treatment of primary biliary cirrhosis with ursodeoxycholic acid, budesonide and fibrates. Dig Dis.

[224] Alvaro D., Gigliozzi A., Marucci L. (2002). Corticosteroids modulate the secretory processes of the rat intrahepatic biliary epithelium. Gastroenterology.

[225] Arenas F., Hervias I., Uriz M. (2008). Combination of ursodeoxycholic acid and glucocorticoids upregulates the AE2 alternate promoter in human liver cells. J Clin Invest.

[226] Prieto J., Qian C., Garcia N. (1993). Abnormal expression of anion exchanger genes in primary biliary cirrhosis. Gastroenterology.

[227] Medina J.F. (2011). Role of the anion exchanger 2 in the pathogenesis and treatment of primary biliary cirrhosis. Dig Dis.

[228] Tanaka H., Makino I. (1992). Ursodeoxycholic acid-dependent activation of the glucocorticoid receptor. Biochem Biophys Res Commun.

[229] Miura T., Ouchida R., Yoshikawa N. (2001). Functional modulation of the glucocorticoid receptor and suppression of NF-kappaB-dependent transcription by ursodeoxycholic acid. J Biol Chem.

[230] Tanaka H., Makino Y., Miura T. (1996). Ligand-independent activation of the glucocorticoid receptor by ursodeoxycholic acid. Repression of IFN-gamma-induced MHC class II gene expression via a glucocorticoid receptor-dependent pathway. J Immunol.

[231] Mitchison H.C., Bassendine M.F., Malcolm A.J. (1989). A pilot, double-blind, controlled 1-year trial of prednisolone treatment in primary biliary cirrhosis: hepatic improvement but greater bone loss. Hepatology.

[232] Yamanishi Y., Nosaka Y., Kawasaki H. (1985). Sterol and bile acid metabolism after short-term prednisolone treatment in patients with chronic active hepatitis. Gastroenterol Jpn.

[233] Volzke H., Baumeister S.E., Alte D. (2005). Independent risk factors for gallstone formation in a region with high cholelithiasis prevalence. Digestion.

[234] Leuschner M., Maier K.P., Schlichting J. (1999). Oral budesonide and ursodeoxycholic acid for treatment of primary biliary cirrhosis: results of a prospective double-blind trial. Gastroenterology.

[235] Rautiainen H., Karkkainen P., Karvonen A.L. (2005). Budesonide combined with UDCA to improve liver histology in primary biliary cirrhosis: a three-year randomized trial. Hepatology.

[236] Angulo P., Jorgensen R.A., Keach J.C. (2000). Oral budesonide in the treatment of patients with primary biliary cirrhosis with a suboptimal response to ursodeoxycholic acid. Hepatology.

[237] Hempfling W., Grunhage F., Dilger K. (2003). Pharmacokinetics and pharmacodynamic action of budesonide in early- and late-stage primary biliary cirrhosis. Hepatology.

[238] Poupon R. (2012). Ursodeoxycholic acid and bile-acid mimetics as therapeutic agents for cholestatic liver diseases: an overview of their mechanisms of action. Clin Res Hepatol Gastroenterol.

[239] Sato H., Macchiarulo A., Thomas C. (2008). Novel potent and selective bile acid derivatives as TGR5 agonists: biological screening, structure-activity relationships, and molecular modeling studies. J Med Chem.

[240] Verma A., Maxwell J.D., Ang L. (2002). Ursodeoxycholic acid enhances fractional calcium absorption in primary biliary cirrhosis. Osteoporos Int.

[241] Fickert P., Wagner M., Marschall H.U. (2006). 24-norUrsodeoxycholic acid is superior to ursodeoxycholic acid in the treatment of sclerosing cholangitis in Mdr2 (Abcb4) knockout mice. Gastroenterology.

[242] Halilbasic E., Fiorotto R., Fickert P. (2009). Side chain structure determines unique physiologic and therapeutic properties of norursodeoxycholic acid in Mdr2−/− mice. Hepatology.

[243] Moustafa T., Fickert P., Magnes C. (2012). Alterations in lipid metabolism mediate inflammation, fibrosis, and proliferation in a mouse model of chronic cholestatic liver injury. Gastroenterology.

[244] Yoon Y.B., Hagey L.R., Hofmann A.F. (1986). Effect of side-chain shortening on the physiologic properties of bile acids: hepatic transport and effect on biliary secretion of 23-nor-ursodeoxycholate in rodents. Gastroenterology.

[245] Trauner M., Claudel T., Fickert P. (2010). Bile acids as regulators of hepatic lipid and glucose metabolism. Dig Dis.

